# Loeffler Endocarditis Causing Heart Failure with Preserved Ejection Fraction (HFpEF): Characteristic Images and Diagnostic Pathway

**DOI:** 10.3390/diagnostics12092157

**Published:** 2022-09-05

**Authors:** Silvia Lupu, Marian Pop, Adriana Mitre

**Affiliations:** 1 M3, Medicala V, ”George Emil Palade” University of Medicine, Pharmacy, Science and Technology of Targu Mures, 540142 Targu Mures, Romania; 2 Cardiology 1 Department, Emergency Institute for Cardiovascular Disease and Heart Transplant of Targu Mures, 540136 Targu Mures, Romania; 3 ME1, ”George Emil Palade” University of Medicine, Pharmacy, Science and Technology of Targu Mures, 540142 Targu Mures, Romania; 4 Radiology and Medical Imaging Department, Emergency Institute for Cardiovascular Disease and Heart Transplant of Targu Mures, 540136 Targu Mures, Romania

**Keywords:** Loeffler endocarditis, imaging, cardiac MRI, echocardiography

## Abstract

We report the case of a 69-year-old female patient in which echocardiography and cardiac magnetic resonance imaging were used to diagnose a patient presenting with heart failure with preserved ejection fraction (HFpEF) due to Loeffler endocarditis. Loeffler endocarditis is an uncommon cause of heart failure with preserved ejection fraction, triggered by eosinophil and lymphocyte infiltration of the endomyocardium, followed by the formation of thrombus in the afflicted area, and eventually fibrosis. This condition is due to an increased number of eosinophils associated with allergies, infections, systemic conditions, as well as malignancies and hypereosinophilic syndrome. Loeffler endocarditis can lead to serious complications, such as progressive heart failure, systemic thromboembolic events, or arrhythmias (including sudden cardiac death).

A 69-year-old female patient presented with dyspnea on moderate exertion, progressively aggravated within the last two years. The patient had no history of angina or syncope. Her medical records showed moderate hypereosinophilia, first documented 5 years before. 

After ruling out a couple of common parasite infections by repeated stool sample analysis, no other causes were investigated. The lady had no history of overt allergy and nor of travel to any exotic destinations. She was treated for depression with escitalopram and lorazepam; both started after the hypereosinophilia was documented. 

On clinical examination, the patient had a blood pressure of 110/80 mmHg, a heart rate of 90 bpm, fine crackles at the bases of both lungs, and bilateral edema of the inferior limbs. 

**Figure 1:**
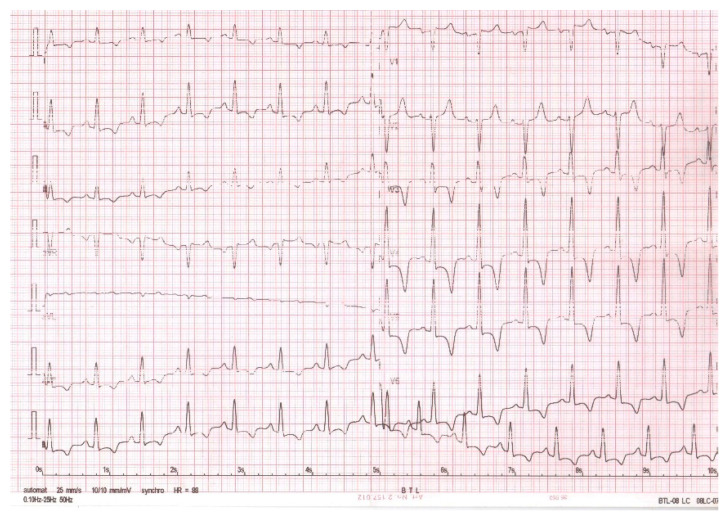
Twelve-lead electrocardiogram—sinus rhythm and deep inverted T waves in most leads (excepting V1, V2, and aVR), and a corrected QT interval of 533 msec.

The initial blood tests showed increased troponin I levels– 155 ng/L (>29 ng/L, Pathfast assay, Medscience Corporation Mitsubishi Chemical Europe), and a blood smear test was performed, yielding moderate hypereosinophilia with no dysmorphic features 4.95 × 10^9^/L (55%). The number of leukocytes (7900/mm^3^), hemoglobin levels (14.3 g/dL), and creatinine were normal (between 0.70 and 0.78 md/dL). 

Echocardiography revealed a normal size left ventricle, with apical obliteration (Figure 2A,B), thickening of the posterior mitral valve and chordae, restricted movement and subsequent mild mitral regurgitation, as well as features of restrictive cardiomyopathy. Based on these characteristics, the diagnosis of Loeffler endocarditis was suspected [1]. 

Cardiac magnetic resonance imaging was further performed. Native and post-contrast (9 mmol gadolinium) images were acquired. bSSFP Cine imaging showed a non-dilated left ventricle with preserved ejection fraction and apical obliteration (Figure 2C and Supplementary Videos S1 and S2). Inversion recovery images 10 min after contrast injection showed sub-endocardial late gadolinium enhancement (LGE) pointing toward the apex and left ventricular apical non-enhancing mass-thrombus (Figure 2D)”. 

The imaging features and the presence of hypereosinophilia were consistent with the diagnosis of Loeffler endocarditis [1,2]. A coronary angio-computed tomography was performed, showing no significant coronary stenosis, a myocardial bridge in the mid-segment of the left anterior descendant artery, and a coronary calcium score of 0. The increase in troponin levels suggested active myocardial inflammation, prompting the initiation of treatment with corticosteroids (methylprednisolone 32 mg/day, starting dose), antihistamines (loratadine 10 mg), as well as loop and antialdosteronic diuretics, an angiotensin-converting enzyme inhibitor, and anticoagulants (acenocumarol and enoxaparin, which was discontinued when therapeutic INR was achieved) [3].

**Figure 2:**
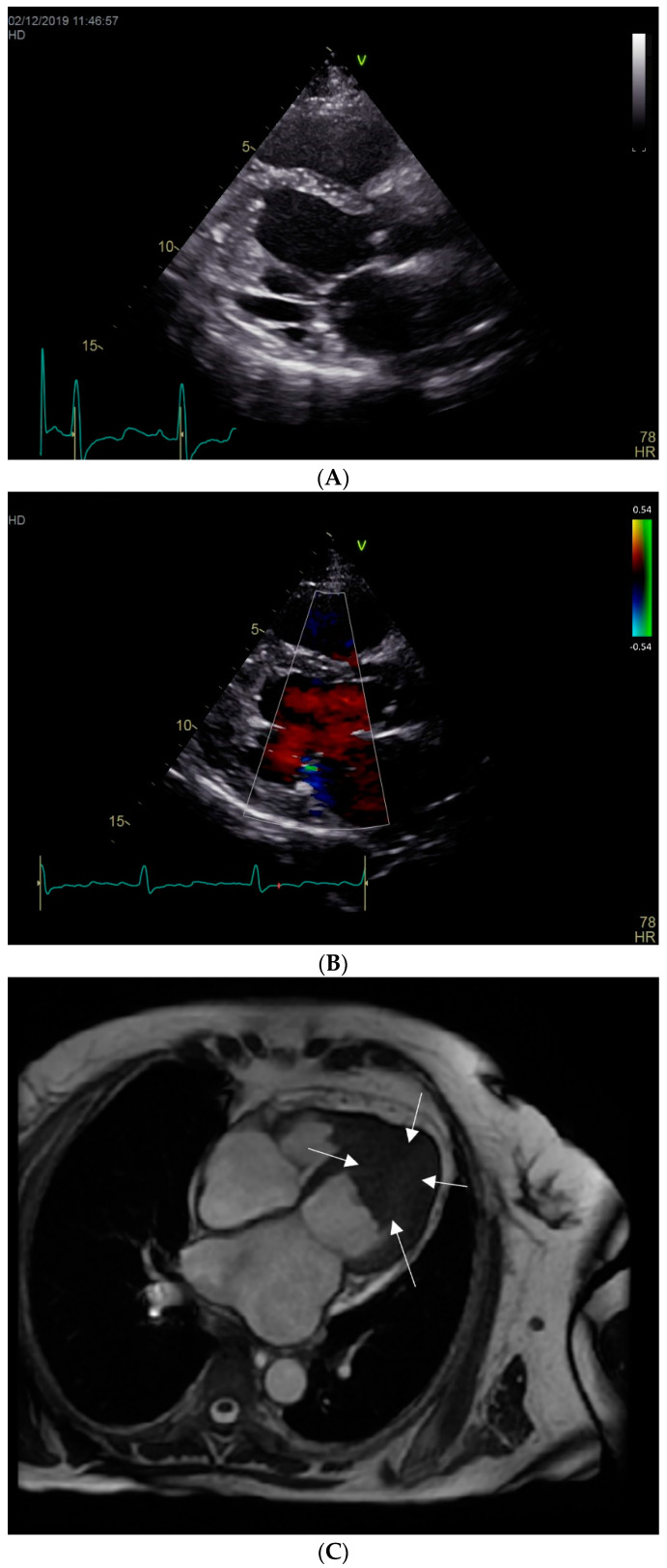

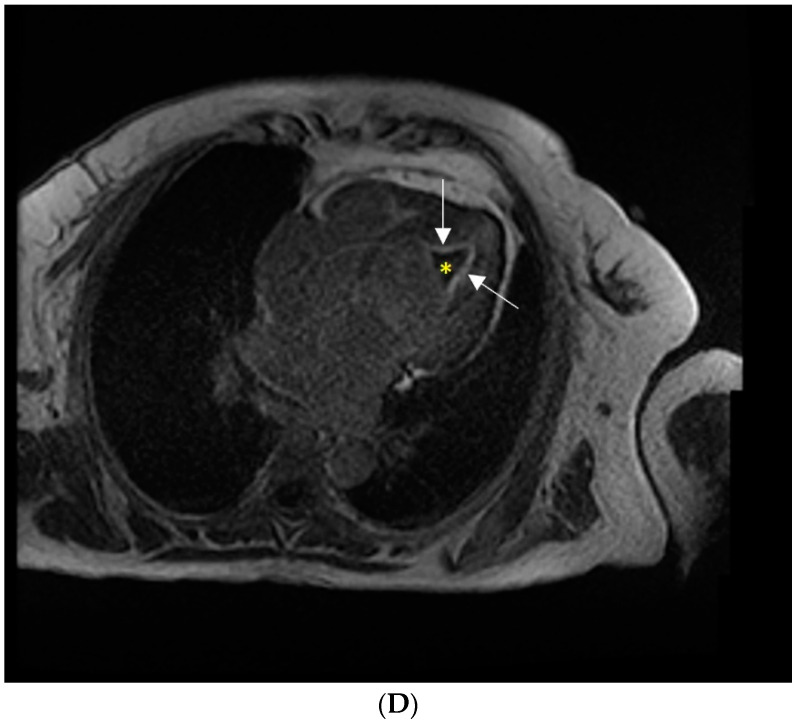
(**A**) Echo Parasternal long axis view showing obliteration of the left ventricular apex. (**B**) Echo Parasternal long axis view: normal-size left ventricle with normal systolic function, increased thickness of the posterior mitral leaflet and chordae, apical obliteration. (**C**) Cardiac MRI: 4 chamber view showing a normal-size left ventricle, with preserved ejection fraction and an apical mass engulfing the papillary muscles. (**D**) Cardiac MRI: inversion recovery images 10 min after contrast injection showing left ventricular apical thrombus (white arrows) and sub-endocardial enhancement (star).

Following treatment, the signs and symptoms of heart failure subsided, and the troponin levels quickly returned to normal levels (19.4 ng/L at discharge). The eosinophil count was normal after a week of treatment. We further attempted to identify the cause of hypereosinophilia. A computer tomography scan ruled out solid malignancy; an area of panacinary emphysema and mild fibrosis at the base of the right lung were visible. Repeated stool samples were negative for parasites. The patient was referred to the internal medicine department to complete the investigations panel for eosinophilia. We considered parasite infection, allergy, rheumatological and hematological disorders as potential causes [3] and scheduled the patient for an appointment with the hematologist to test for JAK2, FIP1L1, CAL-R, and CMPL. However, the patient was non-compliant with recommendations and followed her treatment inconsistently. At the time of discharge, the echocardiography showed the remanence of thrombus, with little change in size. While it is accepted that upon follow-up CMR can show both the decrease in cardiac chamber volumes, the resolution of apical thrombus, and the receding of subendocardial fibrosis, our patient skipped the follow-up visits and was, eventually, lost to follow-up. 

Loeffler endocarditis is a rather rare cause of heart failure, which requires specific treatment and is associated with a very poor prognosis if left untreated. HFpEF in Loeffler endocarditis is due to a change in ventricular compliance, and it shares similar pathophysiology with other restrictive cardiomyopathies; however, its pathophysiology is due to endocardial fibrosis from eosinophilic injuries instead of deposits of amyloid (in amyloidosis), iron (hemochromatosis) or non-caseating granulomas (sarcoidosis). 

Cardiovascular imaging plays a pivotal role in the diagnosis [2,4,5]. While echocardiography features are usually most obvious in the late stages of the disease and may provide insufficient details, cardiac magnetic resonance imaging may be used to identify early changes and to better characterize the endocardium and the presence of thrombus in the late stages [6,7,8]. Although endomyocardial biopsy remains the gold standard for diagnosing the disease, it is an invasive procedure and may sometimes yield falsely negative results [3]. However, cardiac MRI provides compelling evidence of the disease in this patient, such as the presence of subendocardial LGE, and apical thrombus, features that do not emerge in other cardiomyopathies associated with a restrictive pattern, such as sarcoidosis, amyloidosis, or Fabry disease. Although apical hypertrophic cardiomyopathy may exhibit a similar electrocardiographic pattern, the presence of apical thrombus and subendocardial LGE lining the hypertrophied area is unlikely in this condition [4]. 

Every effort should be made to identify the cause of hypereosinophilia and provide targeted treatment.

## Data Availability

Not applicable.

## Supplementary Materials

The following supporting information can be downloaded at: https://www.mdpi.com/article/10.3390/diagnostics12092157/s1, Video S1: CMR 4C view, Video S2: CMR 2C view.
